# Accurate Prediction of the Statistics of Repetitions in Random Sequences: A Case Study in Archaea Genomes

**DOI:** 10.3389/fbioe.2016.00035

**Published:** 2016-06-08

**Authors:** Mireille Régnier, Philippe Chassignet

**Affiliations:** ^1^Inria, Palaiseau, France; ^2^LIX, Ecole Polytechnique, Palaiseau, France

**Keywords:** *K*-mers, combinatorics, probability

## Abstract

Repetitive patterns in genomic sequences have a great biological significance and also algorithmic implications. Analytic combinatorics allow to derive formula for the expected length of repetitions in a random sequence. Asymptotic results, which generalize previous works on a binary alphabet, are easily computable. Simulations on random sequences show their accuracy. As an application, the sample case of Archaea genomes illustrates how biological sequences may differ from random sequences.

## 1 Introduction

This paper provides combinatorial tools to distinguish biologically significant events from random repetitions in sequences. This is a key issue in several genomic problems as many repetitive structures can be found in genomes. One may cite microsatellites, retrotransposons, DNA transposons, long terminal repeats (LTR), long interspersed nuclear elements (LINE), ribosomal DNA, and short interspersed nuclear elements (SINE). In Treangen and Salzberg (2012), it is claimed that half of the genome consists of different types of repeats. Knowledge about the length of a maximal repeat is a key issue for assembly, notably the design of algorithms that rely upon de Bruijn graphs. In re-sequencing, it is a common assumption for aligners that any sequenced “read” should map to a single position in a genome: in the ideal case where no sequencing error occurs, this implies that the length of the reads is larger than the length of the maximal repetition. Average lengths of the repeats are given in Gu et al. (2000). Recently, heuristics have been proposed and implemented (Devillers and Schbath, 2012; Rizk et al., 2013; Chikhi and Medvedev, 2014).

A similar problem has been extensively studied: the prediction of the length of maximal common prefixes for words in a random set. Typical parameters are the background probability model, the size *V* of the alphabet, the length *n* of the sequence, and so on. Deviation from uniformity was studied for a uniform model as early as 1988 (Flajolet et al., 1988). A complexity index that captures the richness of the language is addressed in Janson et al. (2004). A distribution model, valid for binary alphabets and biased distributions, was introduced in Park et al. (2009), the so-called *trie profile* and extended to Patricia tries in Magner et al. (2014). The authors pointed out different “regimes” of randomness and a phase transition, by means of analytic combinatorics (Sedgewick and Flajolet, 2009). It was observed in Jacquet and Szpankowski (1994) that the average length of maximal common prefixes in a random set of *n* words is asymptotically equivalent to the average length of maximal repetitions in a random sequence of length *n*. Sets of words are considered below in the theoretical analysis. A comparison with the distribution of maximal repetitions in random sequences or real Archaea genomic sequences is presented in Section 3.

Our first goal is to extend results of Park et al. (2009) to the case of a general *V*-alphabet, including the special case {*A*, *C*, *G*, *T*} where *V* is 4. A second goal is to compare the results consistency with random data and real genomic data in the finite range.

To achieve the first goal, we rely on an alternative, and simpler, probabilistic and combinatorial approach that is interesting *per se*. It avoids generating functions and the Poissonization–dePoissonization cycle that is used in Park et al. (2009) and it extends to non-binary alphabets. In that case, there is no closed formula for the asymptotic behavior. Nevertheless, the Lagrange multipliers allow to derive it as the solution of an equation that can be computed numerically.

Explicit and computable bounds for the profile of a random set of *n* words are provided. Three domains can be observed. A first domain is defined by a threshold *k* for the length, called the *completion length*: any prefix with a length smaller than this threshold occurs at least twice. This threshold is extremely stable over the data sets and it is highly predictable. A similar phenomenon was observed for a uniform model in Fagin et al. (1979a) and a biased model (Mahmoud, 1992; Park et al., 2009). For larger lengths, some prefixes occur only once. In a second domain, called the *transition phase*, the number of maximal common prefixes is sublinear in the size *n* of the sequence: increasing first, then decreasing slowly, and, finally, dropping rapidly. In the third domain, for a length larger than some *extinction length*, almost no common prefix of that length occurs. Despite the fact that these bounds are asymptotic, a good convergence is shown in practice for random texts when a second-order term is known.

Differences between the model and the observation are studied on the special case of Archaea genomes. A dependency to the GC-content, which is a characteristic of each genome, is exhibited. Regimes and transitions are studied on these genomic data and theoretical results are confirmed, with a drift in the values of transition thresholds. Notably, the length of the largest repetitions is much larger than expected. This difference between the model and the observation arises from the occurrences of long repeated regions.

Section 2 is devoted to Main Results, to be proved in Section 4. First, some notations are introduced; then, an algebraic expression for the expectation of the number of maximal common prefixes in a sequence is derived in Theorem 2.1. Second, this expression is split between two sums that are computable in practical ranges. Then, it is shown that a Large Deviation principle applies. It yields first and second order asymptotic terms, and oscillations, that are provided in Theorem 2.2. A comparison between exact, approximate, and asymptotic expressions is presented in Section 3.

## 2 Main Results

It is assumed throughout this study that sequences and words are randomly generated according to a biased Bernoulli model on an alphabet of size *V*. Let *p*_1_, ⋯, *p_V_* denote the probabilities of the *V* characters χ_1_, ⋯ ,χ*_V_*.

**Definition 2.1**. For any *i* in {1, ⋯ ,*V* }, one notes

$$\beta_{i}=\log\frac{1}{p_{i}}.$$

Additionally,

(1) $$\begin{array}{l} p_{\min}=\min\left\{p_{i};1\leq i\leq V\right\}\;\text{and}\;\alpha_{\min}=\frac{1}{\log\frac{1}{p_{\min}}}=\frac{1}{\max\left(\beta_{i}\right)}; \end{array}$$

(2) $$\begin{array}{l} p_{\max}=\max\left\{p_{i};1\leq i\leq V\right\}\;\text{and}\;\alpha_{\max}=\frac{1}{\log\frac{1}{p_{\max}}}=\frac{1}{\min\left(\beta_{i}\right)}. \end{array}$$

The two values min(*β_i_*) and max(*β_i_*) are different when the Bernoulli model is non-uniform.

### 2.1 Enumeration

**Definition 2.2**. Given *U* a set of words and an integer *k*, *k* ≥ 2, a unique *k*-mer in *U* is a word *wχ_i_* of length *k* such that

1. *w* is a prefix of at least two words in *U*;
2. and *wχ_i_* is a prefix of a single word.

By convention, a unique 1-mer is a character χ*_i_* that is a prefix of a single word.

**Definition 2.3**. Let *U* be a set of *n* words.

For *k* ≥ 1, one denotes *B*(*n, k*) the number of unique *k*-mers in *U*.

One denotes *μ*(*n*, *k* − 1) the expectation of *B*(*n, k*) over all sets of *n* words.

**Remark**: It follows from Definition 2.2 that quantity *B*(*n, k*) is upper bounded by *n*. Observe that, for each random set *U*, it is the sum of a large number – *V^k^* – of correlated random variables. Expectation *μ*(*n, k*) is studied below and compared in Section 3 with *B*(*n, k* + 1).

Profiles of repetitions can be expressed as a combinatorial sum.

**Theorem 2.1**. Given a length *k*, the expectation *μ*(*n, k*) satisfies:

(3) $$\begin{array}{ll} \mu \left(n,k\right)=n\sum_{k_{1}+\cdots k_{v}=k}\left(\begin{array}{c} k\\ k_{1},\cdots \;,k_{V} \end{array}\right)\phi \left(k_{1},\cdots \;,k_{V}\right)\psi_{n}\left(k_{1},\cdots \;,k_{V}\right) & \end{array}$$

where

(4) $$\begin{array}{l} \phi \left(k_{1},\cdots \;,k_{V}\right)=p_{1}^{k_{1}}\cdots p_{V}^{k_{V}} \end{array}$$

(5) $$\begin{array}{l} \psi_{n}\left(k_{1},\cdots \;,k_{V}\right)=\sum_{i=1}^{V}\;p_{i}\left[{\left(1-\varphi \left(k_{1},\cdots ,k_{V}\right)p_{i}\right)}^{n-1}-{\left(1-\varphi \left(k_{i},\cdots ,k_{V}\right)\right)}^{n-1}\right]. \end{array}$$

**Proof**. A word *wχ_i_* is a unique (*k* + 1)-mer iff (i) *w* has length *k* and is the prefix of at least two words, including *wχ_i_*; (ii) *wχ_i_* is not repeated.

Event (i) has probability

$$\begin{array}{l} n\phi \left(k_{1},\cdots \;,k_{V}\right)p_{i}\left[1-{\left(1-\phi \left(k_{1},\;\cdots \;,k_{V}\right)\right)}^{n-1}\right]. \end{array}$$

Event (ii), which is a sub-event of (i), has probability

$$\begin{array}{l} n\phi \left(k_{1},\;\cdots \;,k_{V}\right)p_{i}\left[1-{\left(1-\phi \left(k_{1},\;\cdots \;,k_{V}\right)p_{i}\right)}^{n-1}\right]. \end{array}$$

### 2.2 A Combinatorial Expression

**Definition 2.4**. Given a *k*-mer *w*, let *α* denote $\frac{k}{\log\;n}$ and *k_i_* denote the number of occurrences of character χ_*i*_ in *w*. The *objective function* is

(6) $$\rho \left(k_{1},\cdots \;,k_{V}\right)=\sum_{i=1}^{V}\;\frac{k_{i}}{k}\beta_{i}-\frac{1}{\alpha }.$$

The character distribution (*k*_1_, ⋯ , *k_V_*) of a *k*-mer may be viewed as *barycentric coordinates* for a point $\beta \left(k_{1},\cdots \;,k_{V}\right)=\sum_{i=1}^{V}\;\frac{k_{i}}{k}\beta_{i}$ that lies in $\left[\min\left(\beta_{i}\right);\max\left(\beta_{i}\right)\right]=\left[\frac{1}{\alpha_{\max}};\frac{1}{\alpha_{\min}}\right]$. The order of *β* points on that interval allows for a classification of *k*-mers that is a key to this study.

**Definition 2.5**. A *k*-mer *w* is said

- a common *k*-mer if *ρ*(*k*_1_,…, *k_V_*) < 0;
- a transition *k*-mer if *ρ*(*k*_1_, ⋯ , *k_V_*) ≥ 0 and its ancestor is a common *k*-mer;
- a rare *k*-mer, otherwise.

**Remark**: If *ρ*(*k*_1_, ⋯ , *k_V_*) = 0, the condition on the ancestor is trivially satisfied.

**Definition 2.6**. Given a set *U* of *n* words and an integer *k*, let *D_k_(n)* denote the set of character distributions (*k*_1_, ⋯ , *k_V_*) for rare and transition *k*-mers. Let *E_k_*(*n*) denote the set of character distributions for common *k*-mers.

The set *D_k_*(*n*) is the empty set if *k* < *α_min_* log *n* and is the set of character distributions (*k*_1_, ⋯ , *k_V_*) if *k* > *α_max_* log *n*. Computation of (3) is split among the two sets *D_k_*(*n*) and *E_k_*(*n*). Computations show that the main contribution arises from transition *k*-mers. A probabilistic interpretation will be discussed in 2.4.

**Notation**: Let *S*(*k*) and *T*(*k*) be

(7) $$\begin{array}{l} S\left(k\right)=n\sum_{D_{k}(n)}\;\left(\begin{array}{c} k\\ k_{1}\cdots k_{V} \end{array}\right)\phi \left(k_{1},\cdots \;,k_{V}\right)\psi_{n}\left(k_{1},\cdots \;,k_{V}\right); \end{array}$$

(8) $$\begin{array}{l} T\left(k\right)=n\sum_{E_{k}(n)}\;\left(\begin{array}{c} k\\ k_{1}\cdots k_{V} \end{array}\right)\phi \left(k_{1},\cdots \;,k_{V}\right)\psi_{n}\left(k_{1},\cdots \;,k_{V}\right). \end{array}$$

So *μ*(*n, k*) rewrites

(9) μ(n,k)=S(k)+T(k).

These sums *S*(*k*) and *T*(*k*) can be efficiently computed for moderate *k*, up to a few hundred, approximately. In practice, *α_max_* log *n* is below this threshold for the sizes of actual genomes and for their ordinary GC content value. The simulations in Section 3 show that this estimation is rather tight. Behavior and asymptotic estimates are derived and discussed in the next section.

### 2.3 Asymptotic Estimates

In this section, asymptotic estimates for (3) are derived. First, some characteristic functions are introduced. Then, it is observed that a Large Deviation Principle applies for the combinatorial sums to be computed and asymptotics for the dominating term follow. Amortized terms are also computed. It is shown in Section 3 that this second-order term cannot be neglected in the finite range.

#### 2.3.1 Notations

For general alphabets, asymptotic behavior is a function of the solution of an equation and depends on domains whose bounds are defined below.

**Definition 2.7**. Let (*p_i_*)_1≤_*_i_*_≤_*_V_* be a Bernoulli probability distribution. Let σ_2_ denote $\sum_{i=1}^{V}\;p_{i}^{2}$.

The *fundamental ratio*, noted $\tilde{\alpha }$, is ${\left(\sum_{i}\;p_{i}\log\frac{1}{p_{i}}\right)}^{-1}$.

The *transition ratio*, noted $\bar{\alpha }$, is $\sigma_{2}{\left(\sum_{i}\;p_{i}^{2}\log\frac{1}{p_{i}}\right)}^{-1}$.

The *extinction ratio*, noted *α_ext_*, is $\frac{2}{\log\frac{1}{\sigma_{2}}}$.

**Definition 2.8**. Let *α* be a real value in [*α_min_*, *α_max_*]. Let *τ_α_* be the unique real root of the equation

(10) $$\frac{1}{\alpha }=\frac{\sum_{i=1}^{V}\;\beta_{i}e^{-\beta_{i}\tau }}{\sum_{i=1}^{V}\;e^{-\beta_{i}\tau }}$$

Let *ψ* be the function defined in [*α_min_*, *α_ext_*] as

$$\begin{array}{llll} \alpha_{\min}\leq \alpha \leq \bar{\alpha } & :\psi \left(\alpha \right)=\tau_{\alpha }+\alpha \log\left(\overset{V}{\underset{i=1}{\sum }}\;e^{-\beta_{i}\tau_{\alpha }}\right);\; & & \;\\ \bar{\alpha }\leq \alpha & :\psi \left(\alpha \right)=2-\alpha \log\frac{1}{\sigma_{2}}.\; & & \; \end{array}$$

**Proposition 2.1**. The following property holds

$$\alpha_{\min}\leq \tilde{\alpha }\leq \bar{\alpha }\leq \alpha_{\max}\leq \alpha_{\text{ext}}.$$

Function *ψ* increases on $\left[\alpha_{\min},\tilde{\alpha }\right]$ and decreases on $\left[\tilde{\alpha },\infty \right]$. It satisfies

(11) $$\psi \left(\alpha_{\min}\right)=\psi \left(\alpha_{\text{ext}}\right)=0\;\text{and}\;\psi \left(\tilde{\alpha }\right)=1.$$

**Remark**: Uniqueness of *τ_α_* is shown in Section 4.2. As $\tau_{\bar{\alpha }}=2$, *ψ* is continuous at *α* = $\bar{\alpha }$, with $\psi \left(\bar{\alpha }\right)=2-\bar{\alpha }\log\frac{1}{\sigma_{2}}$.

#### 2.3.2 Asymptotic Results

**Theorem 2.2**. Given a length *α* log *n*, when *n* tends to ∞ the ratio $\frac{\log\;\mu \left(n,\;\alpha \;\log\;n\right)}{\log\;n}$ satisfies:

(12) $$\begin{array}{l} 0\leq \alpha \leq \alpha_{\min}\;\text{or}\;\alpha_{\mathrm{ext}}\leq \alpha \;:\frac{\log\;\mu \left(n,\alpha \log\;n\right)}{\log\;n}\leq 0; \end{array}$$

(13) $$\begin{array}{l} \alpha_{\min}\leq \alpha \leq \alpha_{\mathrm{ext}}\;:\frac{\log\;\mu \left(n,\alpha \log\;n\right)}{\log\;n}∼\psi \left(\alpha \right). \end{array}$$

Moreover, let *ξ* be the function defined in [*α_min_*, *α_ext_*] as $\xi \left(\alpha \right)=\frac{\mu \left(n,\;\alpha \log\;n\right)}{\log\;n}-\psi \left(\alpha \right)$. It satisfies

(14) $$\begin{array}{l} \alpha_{\min}\leq \alpha \leq \bar{\alpha }:\xi \left(\alpha \right)\;∼-\frac{V-1}{2}\frac{\log\left(\alpha \log\;n\right)}{\log\;n}; \end{array}$$

(15) $$\begin{array}{l} \bar{\alpha }\leq \alpha \leq \alpha_{\mathrm{ext}}:\xi \left(\alpha \right)\;∼\frac{\log\left(1-\sigma_{2}\right)}{\log\;n}. \end{array}$$

**Proof**. The key to the proof when *α* ranges in [*α_min_*, *α_max_*] is that *ψ_n_*(*k*_1_, ⋯ *k_V_*) is maximal when *ρ*(*k*_1_, ⋯ *k_V_*) is close to 0. Sum *T*(*k*) satisfies a Large Deviation Principle.

(16) $$\frac{\log\;T\left(\overset{˜}{k}\right)}{k}∼\max\left\{-\overset{V}{\underset{i=1}{\sum }}\;\frac{k_{i}}{k}\log\frac{k_{i}}{k};\rho \left(k_{1},\cdots \;,k_{V}\right)=0\right\}.$$

The maximization problem rewrites as

(17) $$\max\left\{\overset{V}{\underset{i=1}{\sum }}\;\theta_{i}\log\frac{1}{\theta_{i}};\overset{V}{\underset{i=1}{\sum }}\;\theta_{i}=1;\overset{V}{\underset{i=1}{\sum }}\;\beta_{i}\theta_{i}=\frac{1}{\alpha };0\leq \theta_{i}\leq 1\right\}$$

The maximum value is $\tau_{\alpha }+\alpha \log\left(\sum_{i=1}^{V}\;e^{-\beta_{i}\tau_{\alpha }}\right)$ that is reached for the *V*-tuple ${\left(\theta_{i}=\frac{e^{-\beta_{i}\tau_{\alpha }}}{\sum_{i=1}^{V}\;e^{-\beta_{i}\tau_{\alpha }}}\right)}_{1\leq i\leq V}$.

*S*(*k*) satisfies again a Large Deviation Principle when *α* < $\bar{\alpha }$, which yields the asymptotic result in this range. For larger *α*, *S*(*k*) is approximately $\left(1-\sigma_{2}\right)n^{1-\alpha \log\frac{1}{\sigma_{2}}}$ that dominates *T*(*k*).

Details for the proof, including the short and long lengths, are provided in Section 4.

**Remark**: The discussion will depend of the ratio $\alpha =\frac{k}{\log\;n}$. Possible values for *α* range over a *discrete* set as they are constrained to be the ratio of an integer by the log of an integer. An interesting property is that, for any real *α*, the set *T* = {*n* ∈ *N*; *α* log *n* ∈ *N*} is either empty or infinite. Indeed, when *T* is non-empty, it contains all values *n*(α)*^p^* where *n*(α) denotes the minimum value of *T*. It is beyond the scope of this paper to establish the number of other possible solutions.

#### 2.3.3 Domains

Different domains arise from this Theorem, which were observed in Park et al. (2009). Equalities *ψ*(*α_min_*) = 0 and $\psi \left(\bar{\alpha }\right)=2-\bar{\alpha }\log\frac{1}{\sigma_{2}}$ show that there is a continuity between domains.

When *α* lies inside the domain [*α_min_*, *α_ext_*], the ratio $\frac{\log\;\mu \left(n,\alpha \log\;n\right)}{\log\;n}$ is positive and parameters *μ*(*n*, *α* log *n*) are *sub-linear* in the size *n* of the text: some *k*-mers – mostly transition *k*-mers – are unique *k*-mers. Observe that the maximum value for *ψ*(α) is 1. When the Bernoulli model is uniform, this central domain is empty.

When the length is smaller than the *completion length *α*_min_* log *n* or greater than the *extinction length *α*_ext_* log *n*, the ratio $\frac{\log\;\mu \left(n,\;\alpha \log\;n\right)}{\log\;n}$ is negative.

#### 2.3.4 Oscillations

Parameters (*k*_1_, ⋯ , *k_V_*) in the combinatorial sums are integers. As the optimum values (*kθ_i_*)_1≤_*_i_*_≤_*_V_* may not be integers, the practical maximum is a close point on the lattice (*k*_1_, ⋯ , *k_V_*). The difference introduces a multiplicative factor that ranges in $\left[-\log\frac{p_{\max}}{p_{\min}},\log\frac{p_{\max}}{p_{\min}}\right]$. This leads to a small *oscillation* of log *μ*(*n, k*). For large *n*, this contribution to $\frac{\log\;\mu \left(n,k\right)}{\log\;n}$ becomes negligible. As mentioned above, the set of lengths *n* that are *admissible* for a given *α* is very sparse. Nevertheless, an approximate value may be used: for instance, for an integer *k*′, $\begin{array}{l} \frac{1}{k^{\prime }}\log\left\lceil n{\left(\alpha \right)}^{\frac{k^{\prime }}{k}}\right\rceil \end{array}$ is very close to *α*. This oscillation phenomenon was first observed in Nicodème (2005).

#### 2.3.5 Binary Alphabets

Results for binary alphabets in Park et al. (2009) steadily follow from Theorem 2.2. A rewriting of *ψ* leads to alternative expression (18). This *explicit* expression points out the dependency to the distances to *α_min_* and *α_max_*, and the behavior around these points.

**Corollary 2.1**. Assume that the alphabet is binary. Then

(18) $$\psi \left(\alpha \right)=\frac{\alpha }{\log\frac{p_{\max}}{p_{\min}}}\log\left[{s_{\alpha }}^{\frac{1}{\alpha }-\frac{1}{\alpha_{\min}}}+{s_{\alpha }}^{\frac{1}{\alpha }-\frac{1}{\alpha_{\max}}}\right]$$

where

(19) $$s_{\alpha }=\frac{\alpha_{\min}}{\alpha_{\max}}\cdot \frac{\alpha -\alpha_{\min}}{\alpha_{\max}-\alpha }.$$

A similar result holds for DNA sequences when the alphabet is 4-ary and the probability distribution satisfies *p_A_* = *p_T_* and *p_C_* = *p_G_*. Such a distribution is defined by its GC-content *p_G_* + *p_C_*.

### 2.4 A Probabilistic Interpretation

The main contribution to *μ*(*n, k*) arises from *k*-mers with an objective function close to 0, i.e., transition *k*-mers. Such *k*-mers exist in the *transition phase* [*α_min_* log *n*, *α_max_* log *n*] where they coexist with rare or common *k*-mers. Observe that this phase is *shrinked* when the Bernoulli model is uniform, as *p_min_* = *p_max_* and *α_min_* = *α_max_*. Therefore, most unique *k*-mers are concentrated on the two lengths ⌊*α_min_* log *n*⌋ and ⌈*α_min_* log *n*⌉, as observed initially in Fagin et al. (1979b).

Let *k* be some integer in the transition phase. First, the relative contribution of *S*(*k*) and *T*(*k*) to *μ*(*n, k*) varies with the length *k*. For lengths close to *α_min_* log *n*, most words are common and *T*(*k*) dominates *S*(*k*). When *k* increases, the proportion of common words decreases and the relative contribution of *T*(*k*) decreases.

Second, the dominating term in *μ*(*n, k*) arises from transition *k*-mers. Let *w* be a word of length *k*, the character distribution in *w* be (*k*_1_, ⋯ , *k_V_*) and *χ_i_* be some character. The number of words that admit *w* or *wχ_i_* as a prefix fluctuates around the expectations *nϕ*(*k*_1_, ⋯ , *k_V_*) and *nϕ*(*k*_1_, ⋯ , *k_V_*)*p_i_*, respectively. On the one hand, when word *wχ_i_* is a rare word, *nϕ*(*k*_1_, ⋯ , *k_V_*) is less than 1. The smallest *nϕ*(*k*_1_, ⋯ , *k_V_*) is, the less likely the actual number of occurrences of *w* is greater than 2 and the smallest the contribution of *wχ_i_* to *S*(*k*), and *μ*(*n, k*), is. On the other hand, let *wχ_i_* be a common *k* + 1-mer; *w* is a common *k*-mer and then *nϕ*(*k*_1_, ⋯ , *k_V_*) is greater than 1. The largest *nϕ*(*k*_1_, ⋯ , *k_V_*) is, the more likely the word *wχ_i_* is repeated and the smallest the contribution to *T*(*k*), and *μ*(*n, k*), is.

For a short length, i.e., *k* smaller than the completion length *k_min_*, all words are common. In a given sequence, most *k*-mers are repeated at least twice and there is (almost) no unique *k*-mers.

For a large length *k*, i.e., *k* greater than *k_max_*, all words are rare. Nevertheless the number of unique *k*-mers remains sublinear in *n* in the range [*α_max_* log *n*, *α_ext_* log *n*]: the sum of small contributions arising from a large number of possible words is significant.

A folk theorem (Szpankowski, 2001; Jacquet and Szpankowski, 2015) claims that the objective function is concentrated around $\frac{1}{\tilde{\alpha }}-\frac{1}{\alpha }$. Consequently, when *α* = $\tilde{\alpha }$, most *k*-mers are transition *k*-mers and the exponent, the *ψ* function, is maximal.

## 3 Experiments and Analysis

Simulations are presented for random and real data. For each simulation, a suffix tree (Ukkonen, 1995) is built, where each leaf represents a unique *k*-mer. For random cases, the Ukkonen’s insertion step is iterated until a tree with exactly *n* leaves is build. This requires *n* + *k_ins_* insertions of symbols, where *k_ins_* > 0 is relatively small (there is a value of a few dozen in practice for considered *n*). One can observe that the event of having *n* leaves after *n* + *k* − 1 insertions corresponds to the fact that the trailing *k*-mer is unique in the sequence of length *n* + *k* − 1.

Even if a statistical bias exists, with respect to the case of a set of N random words analyzed in previous sections, this bias for respective values on *k* and *n* is below the numeric precision used for tables below.

Then, one simulation that is related to the case of a set of *n* random words, requires the generation of the order of N random symbols from a small alphabet, following a Bernoulli scheme. For this range of *n*, and even in the case of a hundred consecutive simulations, this corresponds to a regular use of a common random number generator (Knuth, 1998).

A first set of simulation deals with the case of random sequences over a binary alphabet, since the results can be compared with previous work. A second set addresses the case of random sequences over a quaternary alphabet {*A*, *C*, *G*, *T*} with a constrained distribution such that probabilities *p_A_* ≈ *p_T_* and *p_C_* ≈ *p_G_* as it is the case for DNA sequences (where the sum *p_C_* + *p_G_* is also known as the GC-content). Results on such random sequences are then compared with the sample biological sequence of an Archaea (*Haloferax volcanii*).

An implementation with a suffix array (Manber and Myers, 1993) allows for a compact representation and an efficient counting (Beller et al., 2013).

### 3.1 Random data

A hundred binary sequences were randomly generated. The number of leaves in each tree was fixed to *n* = 5000000 and the Bernoulli parameter was *p_ma__x_* = 0.7000. Therefore, *p_mi__n_* = 0.3000, $\tilde{p}=0.5429$, and log *n* = 15.4249. The thresholds for *α* and the corresponding lengths *α* log *n* are:

**Table:** 

*α_min_* = 0.8306	$\tilde{\alpha }$ = 1.6370	$\bar{\alpha }$ = 2.0484	*α_max_* = 2.8035	*α_ext_* = 3.6714
*k_min_* = 12.81	$\tilde{k}$ = 25.25	$\bar{k}$ = 31.60	*k_max_* = 43.24	*k_ext_* = 56.63

#### 3.1.1 Statistical Behavior on Random Sets

Throughout experiments, every sample profile for a given sequence fluctuates very little around the expectation.

Table 1 provides experimental results averaged over a hundred binary sequences. Short length with no observed unique *k*-mer is removed. Column 2 gives the mean of *B*(*k* + 1), i.e., the mean number of observed leaves at depth *k* + 1, over the set of a hundred simulations. Columns 3 to 5 give the computed values for *S*(*k*), *T*(*k*), and *μ*(*k*), using the expressions, equations (7–9).

**Table 1:** **Mean profile for 100 random binary sequences**.

	Observed	Predicted			Observed	Asymptotic		
*k*	*B*(*k* *+* 1)	*S*(*k*)	*T*(*k*)	*μ*(*n, k*)	$\frac{\log\;B(k\;+\;1)}{\log\;n}$	ψ(α)	*ψ*(*α*) *+* *ξ*(*α*)
11	0.29	0	0.3	0.3	−0.0803			
12	7.91	0	8.3	8.3	0.1341			
								*k_min_*
13	87.87	0.1	86.9	87.1	0.2902	0.0843	*0.0012*	
14	552.88	1.2	550.3	551.5	0.4094	0.3340	*0.2485*	
15	2456.77	86.6	2366.4	2453.0	0.5061	0.4962	*0.4085*	
16	8269.20	209.4	8069.1	8278.5	0.5848	0.6181	*0.5282*	
17	22516.20	406.1	22097.7	22503.8	0.6497	0.7136	*0.6218*	
18	51085.15	4823.8	46267.2	51091.0	0.7028	0.7897	*0.6960*	
19	99387.01	6636.1	92717.6	99353.7	0.7460	0.8504	*0.7549*	
20	169303.03	37415.5	131882.6	169298.1	0.7805	0.8984	*0.8013*	
21	256358.10	42003.9	214454.4	256458.3	0.8074	0.9357	*0.8370*	
22	349801.23	137615.9	212264.2	349880.1	0.8276	0.9635	*0.8634*	
23	434625.83	134807.6	299824.7	434632.4	0.8416	0.9830	*0.8814*	
24	495572.93	122283.1	373279.8	495562.8	0.8501	0.9949	*0.8919*	
25	522788.19	255284.4	267476.3	522760.7	0.8536	0.9998	*0.8955*	
								$\tilde{k}$
26	513374.76	211204.2	302252.5	513456.7	0.8524	0.9982	*0.8926*	
27	472126.51	315154.7	157087.0	472241.6	0.8470	0.9906	*0.8838*	
28	408946.76	242583.4	166360.3	408943.7	0.8377	0.9772	*0.8692*	
29	335080.05	273441.0	61579.7	335020.7	0.8248	0.9582	*0.8491*	
30	260999.29	198163.4	62712.5	260875.9	0.8086	0.9339	*0.8236*	
31	194100.36	137502.0	56463.1	193965.1	0.7894	0.9043	*0.7930*	
								$\bar{k}$
32	138437.13	122218.3	16090.9	138309.2	0.7675	0.8699	*0.8136*	
33	95017.33	80937.1	14067.8	95004.9	0.7431	0.8346	*0.7783*	
34	63082.67	60397.1	2744.6	63141.7	0.7165	0.7993	*0.7430*	
35	40742.97	38411.9	2368.9	40780.8	0.6882	0.7639	*0.7077*	
36	25679.21	23888.2	1817.4	25705.6	0.6582	0.7286	*0.6724*	
37	15860.59	15622.9	255.8	15878.7	0.6270	0.6933	*0.6371*	
38	9645.84	9455.0	194.2	9649.2	0.5948	0.6580	*0.6018*	
39	5791.32	5772.7	15.9	5788.6	0.5617	0.6227	*0.5664*	
40	3433.87	3426.4	12.1	3438.5	0.5278	0.5874	*0.5311*	
41	2032.57	2027.2	0.4	2027.6	0.4938	0.5520	*0.4958*	
42	1188.84	1189.0	0.3	1189.3	0.4590	0.5167	*0.4605*	
43	692.28	694.8	0.2	695.0	0.4240	0.4814	*0.4252*	
								*k_max_*
44	402.75	405.1	0	405.1	0.3889	0.4461	*0.3899*	
45	233.35	235.7	0	235.7	0.3535	0.4108	*0.3545*	
46	135.42	137.0	0	137.0	0.3182	0.3755	*0.3192*	
47	78.39	79.6	0	79.6	0.2828	0.3401	*0.2839*	
48	44.69	46.2	0	46.2	0.2463	0.3048	*0.2486*	
49	25.35	26.8	0	26.8	0.2096	0.2695	*0.2133*	
50	14.57	15.6	0	15.6	0.1737	0.2342	*0.1780*	
51	8.44	9.0	0	9.0	0.1383	0.1989	*0.1426*	
52	4.76	5.2	0	5.2	0.1012	0.1636	*0.1073*	
53	2.76	3.0	0	3.0	0.0658	0.1282	*0.0720*	
54	1.74	1.8	0	1.8	0.0359	0.0929	*0.0367*	
55	1.02	1.0	0	1.0	0.0013	0.0576	*0.0014*	
56	0.64	0.6	0	0.6	−0.0289	0.0223	−*0.0339*	
								*k_ext_*
57	0.32	0.3	0	0.3	−0.0739	−0.0130	
58	0.18	0.2	0	0.2	−0.1112	−0.0483	
59	0.16	0.1	0	0.1	−0.1188	−0.0836	
60	0.12	0.07	0	0.07	−0.1375	−0.1190	
61	0.08	0.04	0	0.04	−0.1637	−0.1543	
62	0.06	0.02	0	0.02	−0.1824	−0.1896	
63	0.04	0.01	0	0.01	−0.2087	−0.2249	
64	0.04	0.008	0	0.008	−0.2087	−0.2602	

*(*p_max_*; *p_min_*) = (0.7; 0.3)*.

The actual number of leaves *B*(*n*, *k* + 1) is very close to the average value *μ*(*n, k*), and simulations show that this is the general case when (only) a hundred simulations are performed: *μ*(*n, k*) is a very good prediction.

Observed lengths of extinction also show very little variations. In array below, each column gives *n_k_*, the number of sequences out of the one hundred sample set for which the longest repetition had length *k*.

**Distribution of the extinction level for 100 random binary sequences.*p_max_* is 0.7**.

**Table:** 

*k*	51	52	53	54	55	56	57	58	59	60	61	62	63	64
*n_k_*	10	16	13	19	14	14	6	1	1	2	1	1	0	2

In the binary case, the predicted extinction length is between 56 and 57. It is noticeable that, in most cases, the observed depth is slightly smaller than this value. In Table 1, value 0.04 for *μ*(*n*, 61) means that one expects a total of four leaves at depth 60 over one hundred sequences. In that run, exists a total amount of 8.

#### 3.1.2 Quality of Estimates

1. *Tightness of the asymptotic estimates*. Asymptotic estimates (13) given in Column 7 significantly *overestimate* the observed values in Column 6 that is computed directly from Column 2 and *n*. A first conclusion is that first-order asymptotics provide a *poor prediction*: next term is *O*$\left(\frac{1}{\log\;n}\right)$ that goes slowly to 0.
2. *Tightness of the second-order asymptotics*. Second term for the asymptotic *ξ*(*α*) ensures a much better approximation in Column 8.
3. *Growth of asymptotic estimates*. Observed values increase with length until $k=\tilde{k}$ and then decrease. This is consistent with the variation of asymptotic values *ψ*(α).

#### 3.1.3 Dependency to Probability Bias

Thresholds were computed for a given sequence length *n* and various probabilities. The more *p_ma__x_* departs from 0.5, the value for the uniform model, the largest the extinction length is. The completion length, *k_mi__n_*, slightly decreases, while the extinction length significantly increases. Nevertheless, this effect is limited when the largest probability *p_ma__x_* remains in the range [0.5;0.7].

**Dependency of thresholds to *p_max_* for binary alphabets, *n* ***=*** 5,000,000**.

**Table:** 

*p_max_*	*k_min_*	*$\tilde{k}$*	*$\bar{k}$*	*k_max_*	*k_ext_*
0.50	22.25	22.25	22.25	22.25	44.51
0.55	19.32	22.42	22.74	25.80	45.16
0.60	16.83	22.92	24.27	30.20	47.18
0.65	14.69	23.82	27.06	35.81	50.83
0.70	12.81	25.25	31.60	43.25	56.63
0.75	11.13	27.43	38.80	53.62	65.64
0.80	9.58	30.83	50.63	69.13	79.99
0.85	8.13	36.49	71.78	94.91	104.80
0.90	6.70	47.45	116.72	146.40	155.45
0.95	5.15	77.70	259.56	300.72	309.05

### 3.2 Long Repetitions in Archaea Genomes

The experimental data set is the sequence from *Haloferax volcanii DS2 chromosome, complete genome* (Hartman et al., 2010). The alphabet is quaternary. Profile results are shown in Table 2.

**Table 2:** **Profile for the sequence from *Haloferax volcanii* DS2 chromosome, complete genome**.

	Observed	Predicted			
*k*	*B*(*k* *+* 1)	*S*(*k*)	*T*(*k*)	*μ*(*n, k*)
6	4	0	0.05	0.05	
7	1975	0	4e + 02	4e + 02	
8	41349	0	2e + 04	2e + 04	
								*k_min_*
9	178523	781.2	213568.8	214350.1	
10	382032	66858.4	617279.6	684137.9	
11	542386	171711.2	742379.1	914090.3	
								$\tilde{k}$
12	570499	407976.5	215942.2	623918.7	
								$\bar{k}$
13	459330	259860.7	6512.5	266373.2	
								*k_max_*
14	305002	87488.6	0	87488.6	
15	169317	25704.4	0	25704.4	
16	86379	7264.7	0	7264.7	
17	40391	2028.2	0	2028.2	
18	17432	564.1	0	564.1	
19	7866	156.7	0	156.7	
20	3830	43.5	0	43.5	
21	1957	12.1	0	12.1	
22	1229	3.4	0	3.4	
23	910	0.9	0	0.9	
								*k_ext_*
24	733	0.3	0	0.3	
25	617	0.07	0	0.07	
26	561	0.02	0	0.02	
27	492	0.006	0	0.006	
28	446	0.002	0	0.002	
29	436	0.0005	0	0.0005	
30	397	0.0001	0	0.0001	
31	374	1e−05	0	1e−05	
32	359	2e−06	0	2e−06	
33	322	2e−08	0	2e−08	
…	*truncated*	…	*truncated*	…	

Sequence length is *n* = 2847757. The observed symbol frequencies are *p_A_* = 0.1655; *p_C_* = 0.3334; *p_G_* = 0.3330; *p_T_* = 0.1681. Therefore, observed *GC-content* is 0.6664. Parameters for an approximate degenerated quaternary model are *p_A_* = *p_T_* = *p_mi__n_* = 0.1668; *p_C_* = *p_G_* = *p_ma__x_* = 0.3332; $\tilde{p}=0.2645$; and log *n* = 14.8620. The thresholds for the domain are

**Table:** 

*α_min_* = 0.5584	$\tilde{\alpha }$ = 0.7520	$\bar{\alpha }$ = 0.8079	*α_max_* = 0.9099	*α_ext_* = 1.5609
*k_min_* =8.30	$\tilde{k}$ = 11.18	$\bar{k}$ = 12.01	*k_max_* = 13.52	*k_ext_* = 23.20

Statistics on one hundred random sequences with same parameters are shown in Table 3. GC-content is 0.6664. Extinction level is provided in Table 4. Observe first a good match between the observed values, the predicted values for *μ*(*n, k*), and the asymptotic values for random data. As shown for binary alphabets, the observed extinction level for random sequences departs very little from the predicted *k_ext_* level.

**Table 3:** **Mean profile for 100 random degenerated quaternary sequences**.

	Observed	Predicted			Observed	asymptotic		
*k*	*B*(*k* *+* 1)	*S*(*k*)	*T*(*k*)	*μ*(*n, k*)	$\frac{\log B(k+1)}{\log n}$	*ψ*(*α*)	*ψ*(*α*) *+* *ξ*(*α*)
6	0.03	0	0.0	0.0	−0.2359			
7	363.29	0	363.9	363.9	0.3967			
8	21236.17	0	21252.2	21252.2	0.6704
								*k_min_*
9	214371.12	781.6	213574.7	214356.3	0.8260	0.7242	*0.5024*	
10	684344.68	66877.4	617315.1	684192.5	0.9041	0.9280	*0.6956*	
11	914013.67	171742.8	742383.0	914125.8	0.9235	0.9985	*0.7564*
								$\tilde{k}$
12	623870.12	407973.4	215914.6	623888.0	0.8978	0.9655	*0.7147*
								$\bar{k}$
13	266366.73	259826.1	6510.8	266336.9	0.8406	0.8792	*0.8574*
								*k_max_*
14	87424.58	87471.6	0	87471.6	0.7656	0.7930	*0.7711*	
15	25704.95	25698.5	0	25698.5	0.6832	0.7068	*0.6849*	
16	7253.72	7262.9	0	7262.9	0.5981	0.6206	*0.5987*	
17	2025.99	2027.6	0	2027.6	0.5123	0.5344	*0.5125*	
18	565.97	563.9	0	563.9	0.4265	0.4482	*0.4263*	
19	155.90	156.7	0	156.7	0.3397	0.3620	*0.3401*	
20	43.52	43.5	0	43.5	0.2539	0.2758	*0.2539*	
21	12.28	12.1	0	12.1	0.1688	0.1895	*0.1677*	
22	3.06	3.4	0	3.4	0.0753	0.1033	*0.0814*	
23	0.80	0.9	0	0.9	−0.0150	0.0171	−*0.0048*
								*k_ext_*
24	0.28	0.3	0	0.3	−0.0857	−0.0691	−*0.0910*	
25	0.14	0.1	0	0.1	−0.1323	−0.1553	−*0.1772*	

*GC-content is 0.6664*.

**Table 4:** **Distribution of the extinction level for 100 random degenerated quaternary sequences**.

*k*	21	22	23	24	25
*n_k_*	26	42	18	7	7

*GC-content is 0.6664*.

Numerous differences with random data can be observed on real genomes.

Interestingly, the behavior for short lengths and in the transition phase is similar to the random behavior. Observation and prediction have the same order of magnitude. In particular, the number of unique *k*-mers is maximum for length $\tilde{k}$ where observation and prediction coincide. For a real genome and a length *k* smaller than *k_min_*, observed *B*(*n*, *k* + 1) is larger than predicted *μ*(*n, k*). This indicates, at a level *k* + 1 where completion is expected, more leaves in the real trie, more missing words at level *k* + 2. Simultaneously, less internal nodes occur at level *k* + 1 because the total sum is constant and equal to *V^k^*^+1^.

The effect of (non-random) repetitions is more sensible in the decreasing domain. First, the number of unique *k*-mers decreases much more slowly than expected for lengths larger than *k_max_*. A significant gap can be observed around extinction level *k_ext_*. The decrease rate, which was around 0.02–0.04 drops to 0.007 and then becomes even lower. Finally, the extinction level is much larger than the predicted value 23: the largest repetition is 1395 bp long.

To evaluate the contribution of long repetitions, one may erase the longest ones. When a word *w* is repeated, any proper suffix of *w* is also repeated. Consequently, once the longest repeated word is erased, one unique *k*-mer (only) disappears for each length larger than the length of the second largest subsequence (here, 935). The profile remains far from the random profile. This observation is still true if the 10 longest subsequences are erased.

## 4 Combinatorial and Analytic Derivation

### 4.1 Lagrange Multipliers

Lagrange multipliers method allows to maximize an expression under constraints. To compute (17), one sets

(20) $$\begin{array}{l} F\;=\sum_{i=1}^{V}\;\theta_{i}\;\log\;\theta_{i}; \end{array}$$

(21) $$\begin{array}{l} G=\sum_{i=1}^{V}\;\theta_{i}; \end{array}$$

(22) $$\begin{array}{l} H=\sum_{i=1}^{V}\;\theta_{i}\beta_{i}. \end{array}$$

Two constraints are given:

$$G=1\;\text{and}\;H=\frac{1}{\alpha }.$$

An intermediary function *ϕ_α_*(*τ*_1_, ⋯ *τ_V_*) is defined

(23) $$\phi_{\alpha }=F+\lambda_{\alpha }G+\tau_{\alpha }H$$

In order to maximize *ϕ* under these two constraints, *ϕ* function is derived with respect to each random variable *τ_i_*. This yields *V* equations

(24) $$1+\log\theta_{i}+\lambda_{\alpha }+\tau_{\alpha }\beta_{i}=0.$$

Two indices *i_min_* and *i_max_* are chosen that satisfy $\beta_{i_{\min}}\neq \beta_{i_{\max}}$. For instance

$$\begin{array}{lll} \beta_{i_{\min}}=\min{\left(\beta_{i}\right)}_{1\leq i\leq V}=\log\frac{1}{p_{\max}};\; & & \;\\ \beta_{i_{\max}}=\max{\left(\beta_{i}\right)}_{1\leq i\leq V}=\log\frac{1}{p_{\min}}.\; & & \; \end{array}$$

Solving equation (24) with indices *i_min_* and *i_max_* yields

$$\begin{array}{llll} \tau_{\alpha } & =\frac{\log\theta_{i_{\min}}-\log\theta_{i_{\max}}}{\beta_{i_{\max}}-\beta_{i_{\min}}}=\log{\frac{\theta_{i_{\min}}}{\theta_{i_{\max}}}}^{\frac{1}{\beta_{i_{\max}}-\beta_{i_{\min}}}};\; & & \;\\ 1+\lambda_{\alpha } & =\frac{\beta_{i_{\min}}\log\theta_{i_{\max}}-\beta_{i_{\max}}\log\theta_{i_{\min}}}{\beta_{i_{\max}}-\beta_{i_{\min}}}.\; & & \; \end{array}$$

Remaining equations rewrite:

(25) $$\log\theta_{i}=\log\theta_{i_{\min}}+\tau_{\alpha }\left(\beta_{i_{\min}}-\beta_{i}\right).$$

Using the constraint $\sum_{i=1}^{V}\;\theta_{i}=1$ that yields

$$\theta_{i_{\min}}e^{\beta_{i_{\min}}\tau_{\alpha }}\sum_{i=1}^{V}\;e^{-\beta_{i}\tau_{\alpha }}=1,$$

and an expression for $\theta_{i_{\min}}$ follows. Therefore Equation 25 rewrites:

(26) $$\theta_{i}=\frac{e^{-\beta_{i}\tau_{\alpha }}}{\sum_{i=1}^{V}\;\beta_{i}e^{-\beta_{i}\tau_{\alpha }}}.$$

Finally, Equation $\sum_{i=1}^{V}\;\theta_{i}\beta_{i}=\frac{1}{\alpha }$ yields equation (10).

$$\frac{1}{\alpha }=\frac{\sum_{i=1}^{V}\;\beta_{i}e^{-\beta_{i}\tau_{\alpha }}}{\sum_{i=1}^{V}\;e^{-\beta_{i}\tau_{\alpha }}}.$$

For this *V*-tuple

$$\begin{array}{l} \sum_{i=1}^{V}\theta_{i}\log\theta_{i}=-\left(\sum_{i=1}^{V}\;\theta_{i}\beta_{i}\right)\tau_{\alpha }-\left(\sum_{i=1}^{V}\theta_{i}\right)\log\;\left(\sum_{i=1}^{V}\;e^{-\beta_{i}\tau_{\alpha }}\right)=-\frac{\tau_{\alpha }}{\alpha }-\log\left(\sum_{i=1}^{V}e^{-\beta_{i}\tau_{\alpha }}\right). \end{array}$$

### 4.2 Approximation Orders

Derivating the RHS of (10) yields $\frac{\sum_{i\neq j}\;{\left(\beta_{i}+\beta_{j}\right)}^{2}e^{-\left(\beta_{i}+\beta_{j}\right)\tau }}{{\left(\sum_{i}\;e^{-\beta_{i}\tau }\right)}^{2}}$ that is positive. Therefore, for any *α*, the solution to (10) is unique. Moreover, *τ_α_* increases with *α*. Let

(27) $$\begin{array}{ll} \psi_{1}\left(\alpha \right) & =\tau_{\alpha }+\alpha \log\left(\overset{V}{\underset{i=1}{\sum }}\;e^{-\beta_{i}\tau_{\alpha }}\right); \end{array}$$

(28) $$\begin{array}{lll} & \; & \\ \psi_{2}\left(\alpha \right) & =2-\alpha \log\frac{1}{\sigma_{2}}. \end{array}$$

Notably, the solutions *τ_α_* of (10) associated with the four increasing values of *α*: $\left(\alpha_{\min},\tilde{\alpha },\bar{\alpha },\alpha_{\max}\right)$ are (–∞, 1 + 2, + ∞). Computing *ψ* for these values yields (11) and Equality $\psi_{1}\left(\tilde{\alpha }\right)=\psi_{2}\left(\tilde{\alpha }\right)$.

Derivating both expressions yields

(29) $$\begin{array}{ll} \frac{\partial \psi_{1}}{\partial \alpha }\left(\alpha \right) & =\log\left(\overset{V}{\underset{i=1}{\sum }}\;e^{-\beta_{i}\tau_{\alpha }}\right); \end{array}$$

(30) $$\begin{array}{ll} \frac{\partial \psi_{1}}{\partial \alpha }\left(\alpha \right)-\frac{\partial \psi_{2}}{\partial \alpha }\left(\alpha \right) & =\log\left(\frac{1}{\sigma_{2}}\overset{V}{\underset{i=1}{\sum }}\;e^{-\beta_{i}\tau_{\alpha }}\right). \end{array}$$

Both derivatives are monotone functions of *τ_α_*. In equation (30), derivative is 0 when $\alpha =\bar{\alpha }$. Therefore, *ψ* is the maximum of the two values *ψ*_1_ and *ψ*_2_ over the interval [*α_min_*, *α_max_*]. The former equation is 0 if *α* = $\tilde{\alpha }$. Therefore, *ψ* is maximum when *α* = $\tilde{\alpha }$.

### 4.3 Approximations

#### 4.3.1 Short Lengths

Assume that *k* ≤ *α_min_* log *n*. Each term *ϕ*(*k*_1_, ⋯ , *k_V_*) is lower bounded by $p_{\min}^{k}=n^{\alpha \log p_{\min}}=n^{-\frac{\alpha }{\alpha_{\min}}}$. Each term *ψ_n_*(*k*_1_, ⋯ , *k_V_*) is trivially bounded by $e^{-n^{1-\frac{\alpha }{\alpha_{\min}}}}$ that is upper bounded by 1 and *nψ_n_*(*k*_1_, ⋯ , *k_V_*) tends to 0 when *n* goes to ∞. As $\sum \;\left(\begin{array}{c} k\\ k_{1},\cdots k_{v} \end{array}\right)\phi \left(k_{1},\cdots \;,k_{V}\right)=1$, the ratio $\frac{\log\;\mu \left(n,\;k\right)}{\log\;n}$ is negative.

#### 4.3.2 Moderate and Large Lengths

For a length *k* in the transition domain [*α_min_* log *n*, *α_max_* log *n*], the objective function may be either positive or negative. When *k* > *α_max_* log *n*, set *E_k_*(*n*) is empty and *μ*(*n, k*) reduces to *S*(*k*).

The maximum *M* among the terms $e^{k\left(-\sum_{i}\;\frac{k_{i}}{k}\log\frac{k_{i}}{k}-\frac{1}{k}\log\;n\phi \left(k_{1},\cdots \;,k_{V}\right)\right)}$ in *T*(*k*) is reached when *ρ*(*k*_1_,  ⋯ , *k_V_*) is 0. Due to the exponential decrease of $e^{-n\phi \left(k_{1},\cdots \;,k_{V}\right)}$ when *nϕ*(*k*_1_, ⋯ , *k_V_*) ≥ 1, $\frac{T\left(k\right)}{k}$ is upper bounded. Computation of log *M* is done with Lagrange multipliers, as explained above.

Computation of *S*(*k*) relies on the local development of *ψ_n_*(*k*_1_, ⋯ , *k_V_*), that is *n*(1–*σ*_2_)*ϕ* (*k*_1_, ⋯ , *k_V_*). *S*(*k*) rewrites ${\sigma_{2}}^{k}\tilde{S}\left(k\right)+\left(S\left(k\right)-{\sigma_{2}}^{k}\tilde{S}\left(k\right)\right)$ where $\tilde{S}\left(k\right)=\sum_{\rho \left(k_{1},\cdots \;,k_{V}\right)\leq 0}\;\left(\begin{array}{c} k\\ k_{i} \end{array}\right){\left(\frac{p_{1}^{2}}{\sigma_{2}}\right)}^{k_{1}}\cdots {\left(\frac{p_{V}^{2}}{\sigma_{2}}\right)}^{k_{V}}$. This sum satisfies a Large Deviation Principle when $\rho \left(k_{1},\cdots \;,k_{V}\right)+\frac{1}{\alpha }\geq \frac{1}{\tilde{\alpha }}$, or *α* < $\tilde{\alpha }$. In this range, $\frac{\tilde{S}\left(k\right)}{k}∼\max\left\{-\sum_{i=1}^{V}\;\frac{k_{i}}{k}\log\frac{k_{i}}{k}\right\}$, which was shown to be *ψ*(α).

When *α* > $\tilde{\alpha }$, sum S˜(k) rewrites $1-\bar{S}\left(k\right)$ where

$$\bar{S}\left(k\right)=\underset{\rho \left(k_{1},\cdots \;,\;k_{V}\right)+\frac{1}{\alpha }<\frac{1}{\tilde{\alpha }}}{\sum }\;\left(\begin{array}{c} k\\ k_{i} \end{array}\right){\left(\frac{p_{1}^{2}}{\sigma_{2}}\right)}^{k_{1}}\cdots {\left(\frac{p_{V}^{2}}{\sigma_{2}}\right)}^{j_{V}}.$$

This sum satisfies a Large Deviation Principle and

$$\frac{\bar{S}\left(k\right)}{k}∼\max\left\{-\sum_{i=1}^{V}\;\frac{k_{i}}{k}\log\frac{k_{i}}{k}+\sum_{i=1}^{V}\;\frac{k_{i}}{k}\log\frac{p_{i}^{2}}{\sigma_{2}}\right\}.$$

As $\sum_{i=1}^{V}\;\frac{k_{i}}{k}\;\log\frac{p_{i}^{2}}{\sigma_{2}}=-\frac{2}{\alpha }+\log\frac{1}{\sigma_{2}}$, this maximum is

$$-\frac{1}{\alpha }\left[2-\alpha \log\frac{1}{\sigma_{2}}-\psi \left(\alpha \right)\right]$$

that is negative.

### 4.4 Binary Case

Barycentric coordinates of *α* are unique. Indeed, equation (10) reduces to a linear equation on the variable $e^{-\left(\beta_{2}-\beta_{1}\right)\tau }$

$$\frac{1}{\alpha }=\frac{\beta_{1}+\beta_{2}e^{-\left(\beta_{2}-\beta_{1}\right)\tau }}{1+e^{-\left(\beta_{2}-\beta_{1}\right)\tau }}$$

where $\beta_{2}-\beta_{1}=\beta_{\min}-\beta_{\max}=\log\frac{p_{\max}}{p_{\min}}$. Therefore, $e^{-\left(\beta_{2}-\beta_{1}\right)\tau }=\frac{1-\alpha \beta_{1}}{\alpha \beta_{2}-1}$. Finally

$$\tau_{\alpha }=\frac{1}{\log\frac{p_{\max}}{p_{\min}}}\log\frac{\alpha \beta_{2}-1}{1-\alpha \beta_{1}}=\frac{1}{\log\frac{p_{\max}}{p_{\min}}}\log\frac{\frac{1}{\alpha_{\min}}-\frac{1}{\alpha }}{\frac{1}{\alpha }-\frac{1}{\alpha_{\max}}}.$$

Function *ψ* rewrites, in the binary case:

$$\psi_{\alpha }=\tau_{\alpha }=\alpha \log e^{-\frac{1}{\alpha }\tau_{\alpha }}\left(e^{-\left(\beta_{1}-\frac{1}{\alpha }\right)\tau_{\alpha }}+e^{-\left(\beta_{2}-\frac{1}{\alpha }\right)\tau_{\alpha }}\right).$$

Observing that $e^{-\left(\beta_{2}-\beta_{2}\right)\tau_{\alpha }}=s_{\alpha }$ and changing variable *τ_α_* into (*β*_2_ − *β*_1_) yields $e^{-\left(\beta_{1}-\frac{1}{\alpha }\right)\tau_{\alpha }}={s_{\alpha }}^{-\left(\frac{1}{\alpha_{\min}}-\frac{1}{\alpha }\right)}$ and $e^{-\left(\beta_{2}-\frac{1}{\alpha }\right)\tau_{\alpha }}={s_{\alpha }}^{-\left(\frac{1}{\alpha_{\max}}-\frac{1}{\alpha }\right)}$.

## 5 Conclusion

This paper describes the behavior of the number of unique or repeated *k*-mers in a random sequence, on a general alphabet. Derivation relies on a combination of analytic combinatorics and on Lagrange multipliers. It simplifies an approach provided for binary alphabets and allows to address larger alphabets, including the quaternary alphabets, such as DNA alphabet. Precise asymptotic estimates are provided and a probabilistic interpretation is given. They are validated on random simulated data and shown to be valid in the finite range. Therefore, they provide a valuable tool to estimate a suitable read length for assembly purposes and tune parameters for assembly algorithms. Real genomes significantly depart from the random behavior for long repetitions. The general shape of the trie profile is observed, with a maximum of the number of unique *k*-mers at the expected length. However, for real genomes, a number of very short *k*-mers are missing and, on the contrary, one observes a number of very long repetitions. Besides these events, the behaviors are rather similar.

In the future, it is worth extending the method to generalized Patricia tries, Markov models and approximate repetitions.

## Author Contributions

Both authors contributed equally.

## Conflict of Interest Statement

The authors declare that the research was conducted in the absence of any commercial or financial relationships that could be construed as a potential conflict of interest.
